# Aldol condensation of refluxing acetone on CaC_2_ achieves efficient coproduction of diacetone alcohol, mesityl oxide and isophorone†

**DOI:** 10.1039/c8ra05965a

**Published:** 2018-08-30

**Authors:** Xuebing Xu, Hong Meng, Yingzhou Lu, Chunxi Li

**Affiliations:** State Key Laboratory of Chemical Resource Engineering, Beijing University of Chemical Technolog Beijing 100029 PR China; College of Chemical Engineering, Beijing University of Chemical Technology Beijing 100029 PR China; Beijing Key Laboratory of Energy Environmental Catalysis, Beijing University of Chemical Technology Beijing 100029 PR China Licx@mail.buct.edu.cn

## Abstract

Efficient production of diacetone alcohol (DAA), mesityl oxide (MO) and isophorone (IP) is important for the high value utilization of acetone. For this, a novel process is proposed for the aldol condensation of acetone by refluxing on CaC_2_, whereby 95% of total selectivity of (DAA, MO and IP) and 85% of acetone conversion are achieved simultaneously under mild conditions (56 °C, 100 kPa). This result is superior to the previously reported ones, which may be ascribed to the extremely high catalytic capacity of CaC_2_ for the aldol condensation and instant separation of the target products from the catalyst to suppress the unwanted successive condensations. Moreover, the target products may be adjusted by operation conditions. Lower temperature and higher reflux rate favour the production of DAA and MO; MO and IP are favoured otherwise. The present process integrates several functions into one unit, *i.e.* the basic catalysis of calcium carbide, aldol condensation of acetone, instant distillation separation of the condensates, and the hydrolysis of CaC_2_ to C_2_H_2_, which makes it feasible to coproduce C_2_H_2_ and high value derivatives of acetone simultaneously.

## Abstract 2

The refluxing reaction of acetone on CaC_2_ realizes an efficient coproduction of diacetone alcohol, mesityl oxide, isophorone and C_2_H_2_.

## Graphical abstract

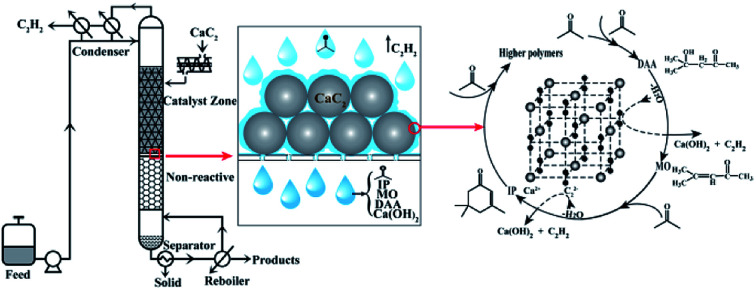

## Introduction

Aldol condensation of acetone is an important reaction in the production of many valuable chemicals, *e.g.* diacetone alcohol (DAA), mesityl oxide (MO) and isophorone (IP). DAA is a low volatile green solvent and is widely used as an extractant, preservative, and anti-freeze agent.^1^ Besides, DAA is an intermediate in the production process of methyl isobutyl ketone and methyl isobutyl alcohol.^2,3^ MO may be used either as a solvent in preparing polyvinyl chloride, polymer resin, dye and ink^4^ or as a feedstock in many organic synthesis.^5^ Similarly, IP is a good solvent for inks, lacquers, gums and coating materials,^6^ as well as a reactant for polyurethanes, pharmaceuticals, fragrances and other commercial chemicals.^7–9^

Currently, NaOH or KOH is used as a catalyst for the liquid phase condensation of acetone,^10^ which is confronted with great challenges such as corrosiveness, hard recycling and low catalytic activity. In order to overcome these problems, a better catalyst needs to be developed. Teissier *et al.*^11^ reported the liquid-phase condensation of acetone with magnesium aluminium double oxide as a solid catalyst, by which 38% of conversion and 67% of total selectivity for MO, DAA and IP was achieved at 200 °C and 2.5 MPa. The catalytic performance of many solid bases such as Ca(OH)_2_, Ba(OH)_2_, Sr(OH)_2_ (ref. 12) and MgAl hydrotalcites^13,14^ were investigated. It was found that the catalyst should have more strong basic sites, larger surface area, smaller size, and better dispersion. For example, when Mg–Al layered double hydroxides (LDH) are supported on multi-walled carbon nanotubes, their catalytic activity triples in comparison with the pristine LDH at 273 K with 100% of selectivity for DAA and less than 10% of conversion of acetone.^14^ To further increase the conversion of acetone, many researchers had recourse to the high temperature vapor-phase reaction of acetone. León *et al.*^15^ studied the catalytic activity of MgO with high surface area, and achieved 33% of conversion and 67% of overall selectivity for MO, DAA and IP at 523 K. Liu *et al.*^16^ demonstrated that Cr, Zr-doped LDHs have more basic sites and higher basicity, and accordingly better catalytic activity than those of the pristine LDH, achieving 36.8% of conversion at 513 K with selectivity of 72.5% for IP and 8.9% for MO. Until now, many basic oxides or oxide composites have been used for the vapor-phase condensation of acetone, *e.g.* molecular sieve,^17^ CaO–SnO_2_,^18^ MgO,^15,19^ Mg–Zr and Mg–Al mixed oxide,^14,16,20^ TiO_2_,^21,22^ (VO)_2_P_2_O_7_ (ref. 23) and even carbon materials.^24^ Among them, Mg–Al mixed oxides showed better catalytic performance.

As a whole, the metal oxide based catalysts show lower catalytic activity due to their weak basicity, and the conversion of acetone is quite low even at high temperatures (200–400 °C). It is well known that the aldol condensation is a base catalysed process, and the stronger the basicity of the catalyst is, the higher the catalytic activity is. Therefore, in order to realize an efficient aldol condensation under mild conditions, we have to explore new catalysts with much stronger Lewis basicity than the metal oxides. In this regard, calcium carbide might be an excellent candidate due to its extremely strong Lewis basicity,^25^ as justified by our previous research.^26^ The catalytic activity of CaC_2_ is superior to all basic catalysts reported in terms of the conversion of acetone. In fact, this process unified the basic catalysis and hydrolysis of CaC_2_ processes, which promoted the condensation of acetone along with the quantitative reclamation of acetylene. The conversion of acetone exceeded 80% within 2 h at 150 °C. However, the reaction selectivity for DAA, MO and IP was not satisfactory due to their successive condensation, forming 50% of highly condensed derivatives of acetone. In order to fully use the catalysis of CaC_2_ and improve the selectivity for DAA, MO and IP, it is necessary to separate them instantly from the catalyst so as to avoid their successive condensation. This may be achieved by catalytic distillation (CD) process considering their large difference in boiling points, *i.e.* acetone (56 °C), DAA (167.9 °C), MO (129.7 °C) and IP (215.2 °C).

CD is such a process that combines a heterogeneous catalytic reaction and product separation into a unified distillation column, where the packing is the catalyst itself. This process is especially suitable for consecutive reactions and those reactions restricted by equilibrium, and thus has been commercially used for the production of many ethers^27,28^ and esters,^29^ such as methyl *tert*-butyl ether, ethyl *tert*-butyl ether, methyl acetate and butyl acetate. For the aldol condensation of acetone, CD process has also been studied using ion exchange resins^30,31^ and MgO-coated saddles^32^ as catalyst. For example, using Amberlite IRA-900 resin as catalyst,^33^ the concentration of DAA in CD experiment was twice the value in conventional fixed bed reactors. Based on this, as an efficient catalyst for the aldol condensation of acetone, CaC_2_ can be packed in a CD column to avoid successive condensation. The process combined the aldol condensation of acetone and the production of C_2_H_2_ together in a CD column; the reaction was performed with refluxing acetone on CaC_2_ surface. Herein, CaC_2_ is not only a catalyst but also a reactant in this process, being an extension of CD and new application of CaC_2_. And the new uses of CaC_2_ has attracted extensive attention in recent years, such as in preparation of carbon materials,^34–36^ polyynes,^37^ di-substituted alkynes,^38^ vinyl ethers,^39^ propargyl alcohols^40^ and so on. The present process provides a new application of super-basicity of CaC_2_ in aldol condensation and may be viable for other condensation reactions.

In this work, we have studied the influence of temperature on the conversion of acetone and selectivity of DAA, MO and IP for the liquid-phase condensation of acetone on CaC_2_. Then, the influence of reaction temperature and separation efficiency on the products selectivity was studied. Finally, a schematic process with CaC_2_ was proposed for the efficient conversion of acetone to DAA, MO and IP, which is of practical significance for the high value utilization of acetone.

## Experimental

### Materials

Acetone (AR, ≥99.5%) was purchased from Beijing Chem. Works (China). CaC_2_ (industrial grade, 75%) was purchased from Tianjin FuChen Chem. Reagent Factory (China). Ethanol absolute (AR, ≥99.7%) was purchased from Tianjin Damao Chem. Reagent Factory (China).

### One-pot liquid phase condensation of acetone at different temperatures

10 g of acetone and 4 g of CaC_2_ was added to an autoclave. The autoclave was placed in a thermostatic oil bath for 10 h at a specified temperature (30 °C, 60 °C, 70 °C, 130 °C) and then cooled to room temperature. The products were first separated *via* filtration, and then the liquid and solid samples were analyzed respectively.

### The refluxing reaction of acetone on CaC_2_ surface

About 100 g of acetone was added to a 250 mL three-necked round-bottom flask and 40 g of granular CaC_2_ was put on the porous glass support of the distillation column, for details please see Fig. S1 (ESI).† The flask was heated to the boiling state of the liquid by an electric heater and the temperature of the liquid and vapor in the column was recorded by two thermocouples. In the boiling state, the acetone vapor went up to the Allihn condenser through a side pipe, and condensed therein to its boiling liquid and dropped back to the surface of CaC_2_ catalyst. In order to reduce the evaporation loss of acetone, we used a 40% glycol aqueous solution (−10 °C) as the coolant of the condenser connected to a cryogenic thermostatic bath (DFY-10/10, Shanghai Lingbiao), and all the connecting joints were securely sealed by PTFE sealing tape and parafilm. The condensation reaction occurred constantly on the surface of CaC_2_. The as-formed products were removed instantly from the catalyst by the refluxing acetone to the flask, and remained there in the whole reaction process due to their much lower volatility than acetone and easier condensation in the side pipe by air cooling. As such, deep condensation of acetone can be avoided significantly. Once steady reflux had been established, the temperatures in the flask and the reaction zone were recorded and then the start timing for the reaction was recorded. A syringe was used for sampling the liquid mixture at set time intervals. The resulting water from condensation of acetone reacts with CaC_2_ immediately, forming C_2_H_2_ and Ca(OH)_2_. The acetylene gas was collected by a draining method from the top of the condenser, and the Ca(OH)_2_ formed on the surface of CaC_2_ was washed away dynamically by the down-flowing liquid.

### Analysis

Liquid sample was taken by a syringe and diluted with anhydrous ethanol. The resulting solution was analyzed by gas chromatography (GC, Shimadzu GC-2010, Japan) equipped with a flame ionization detector and a 30 m-long FFAP capillary column. Nitrogen (99.999% in purity) was used as the carrier gas at a constant flow of 3.0 mL min^−1^. The GC oven temperature was programmed from 50 °C (held for 3 min) to 230 °C (held for 5 min) at 18 °C min^−1^, and 0.5 μL of sample was injected into the split mode when the injector temperature was set at 250 °C. The concentrations of acetone and the products were analyzed by internal standard curve method; the standard curves are shown in Fig. S2,† and the conversion was defined as the percentage of acetone converted. The selectivity for each product was expressed as the amount of the specified product divided by the amount of all products and multiplied by 100.

## Results and discussion

### Influence of temperature on the one-pot condensation reaction

**Fig. 1:**
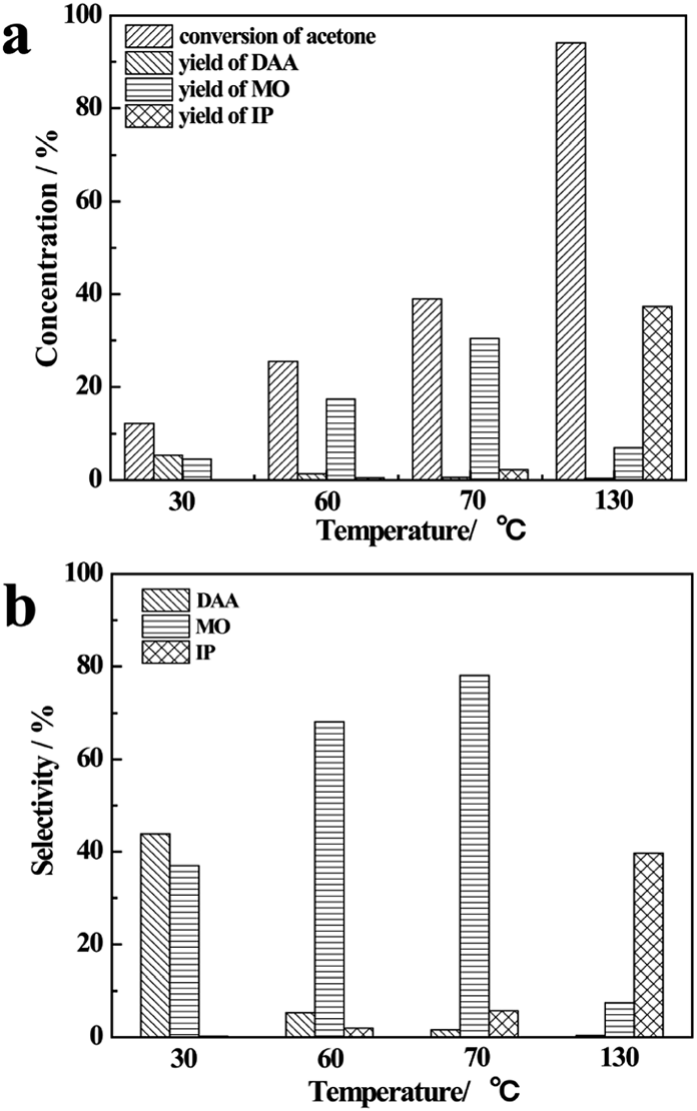
Influence of temperature on concentration (a) and selectivity (b).

The results of reaction of acetone and CaC_2_ at different temperatures are presented in Fig. 1.

As the temperature rises from 30 °C to 130 °C, the conversion of acetone and the yield of IP increase remarkably, and the yield of DAA decreases greatly, while the yield of MO increases from 30 °C to 70 °C at first and then decreases. At 30 °C, the conversion of acetone is only about 10%; DAA and MO are the main products with their total selectivity being about 80%. As the temperature rises to 60 °C and 70 °C, the conversion of acetone and the yield of MO increase remarkably, while the yield of DAA decreases to less than 1%, making MO the main product. As the temperature rises further to 130 °C, the conversion of acetone reaches 95%, IP becomes one of the main products along with large amount of oligomers, and the total selectivity of DAA, MO and IP is relatively low, as shown in Fig. 1(b).

**Scheme 1:**
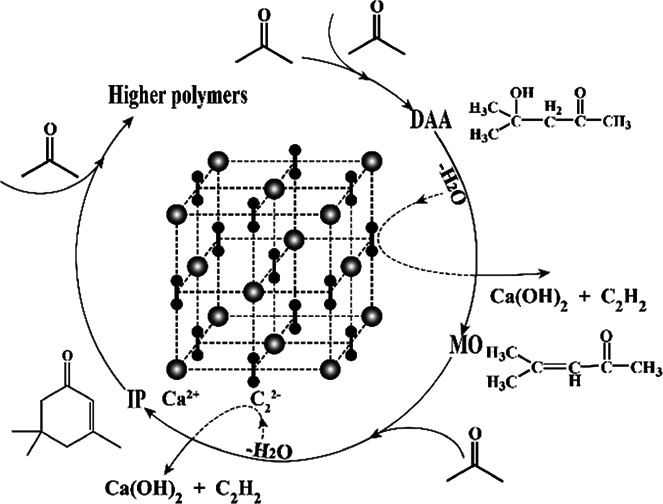
The aldol condensation process of acetone under basic catalysis.

This finding is understandable by referring to the derivative relationship among acetone, DAA, MO and IP through a consecutive reaction of acetone, as presented in Scheme 1. Clearly, DAA is the first product formed *via* condensation of two acetone molecules. It is converted to MO at higher temperature through an elimination reaction under basic catalysis. MO may be converted to IP or oligomers by further condensation, dehydration and Michael addition *via* subsequent aldol condensation.^26^ It is worth noting that CaC_2_ herein is not only a strong catalyst but also a strong dehydrating agent, which promotes the aldol condensation as well as elimination reactions significantly and produces C_2_H_2_ simultaneously. In short, temperature has a profound influence on the consecutive reactions of acetone and product selectivity. High temperature can lead to higher conversion of acetone and yield of IP, along with formation of oligomer byproducts and lower selectivity of DAA and MO. In order to improve the selectivity of DAA, MO and IP, it is necessary to separate them from CaC_2_, which prevents their further conversion to oligomers. Therefore, the product distribution of DAA, MO and IP is highly dependent on temperature.

### Product selectivity for the refluxing reaction of acetone on CaC_2_ surface at 56 °C

**Fig. 2:**
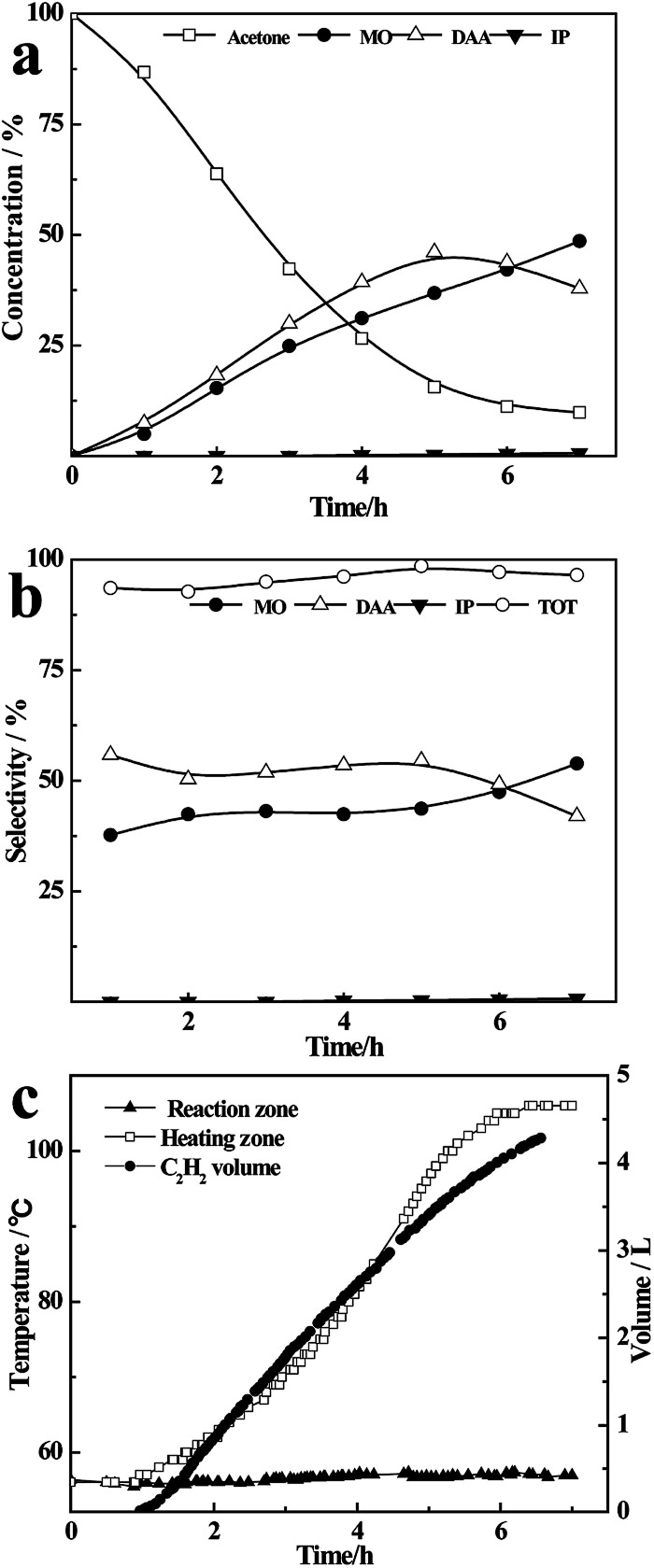
The concentration (a), selectivity (b) and temperature range and C_2_H_2_ volume (c) at the boiling point of acetone.

As shown in Fig. 2(a), the concentration of acetone in the liquid mixture decreases rapidly from 100% to 15% in 5 h, along with an increasing content of DAA and MO. After that, the concentration of acetone only decreases ∼5% within 2 h, accompanying the conversion of DAA to MO. This may be ascribed to the low concentration of acetone and high boiling temperature of the liquid mixture at the final stage, which increase the concentration of DAA in the vapour phase and accordingly its dehydration to MO, resulting in a mixture of acetone (9.8%), DAA (37.9%), MO (48.6%), IP (0.6%) and oligomers (3.1%). As shown in Fig. 2(b), the total selectivity of DAA, MO and IP is more than 95% in the whole reaction process, and the selectivity of DAA is a little higher than that of MO within 6 h, while the yield of IP is basically negligible.

As shown in Fig. 2(c), the liquid temperature in the flask rises from 56 °C to 110 °C as the reaction proceeds, but the temperature in the reaction zone remains at 56 °C in the whole process. Meanwhile, the volume of C_2_H_2_ increases quickly with time, accompanying the consumption of CaC_2_ (Fig. S3†), and finally reaches 4300 mL, which is approximate to the theoretical value of 4380 mL. The results for the refluxing and one-pot reactions under the same heating temperature and masses of reactants are compared in Fig. S4 (ESI).† Obviously, the conversion rate of acetone in the one-pot reaction increases more quickly than that in the refluxing reaction (see Fig. S4(a)†). The total selectivity in the one-pot reaction decreases significantly with time with only about 30% of DAA, MO and IP, being much lower than that in refluxing reaction (see Fig. S4(b)†). This can be attributed to the rising temperature with time in the one-pot reaction (see Fig. S4(c)†) and further condensation of the resultant DAA, MO and IP catalysed by CaC_2_. The results indicate that the refluxing reaction of acetone on CaC_2_ realizes an excellent control of reaction temperature on catalyst and instant separation of the heavier intermediates from the catalyst by the refluxing acetone, resulting in a higher selectivity of DAA, MO and IP.

### Product selectivity for the refluxing reaction of acetone on CaC_2_ at higher temperatures (∼90–115 °C)

In order to enhance the selectivity for MO and IP, it is necessary to conduct the aldol condensation at a higher temperature, which can be facilely achieved in practice in a pressure column. Herein, we conducted the reaction in the temperature range of ∼90–115 °C by using a liquid mixture (100 g toluene and 10 g acetone) as the starting reactants, and 8 g of CaC_2_ catalyst in the distillation column. The flask was fixed in an electric heating sleeve at a constant temperature of 350 °C.

**Fig. 3:**
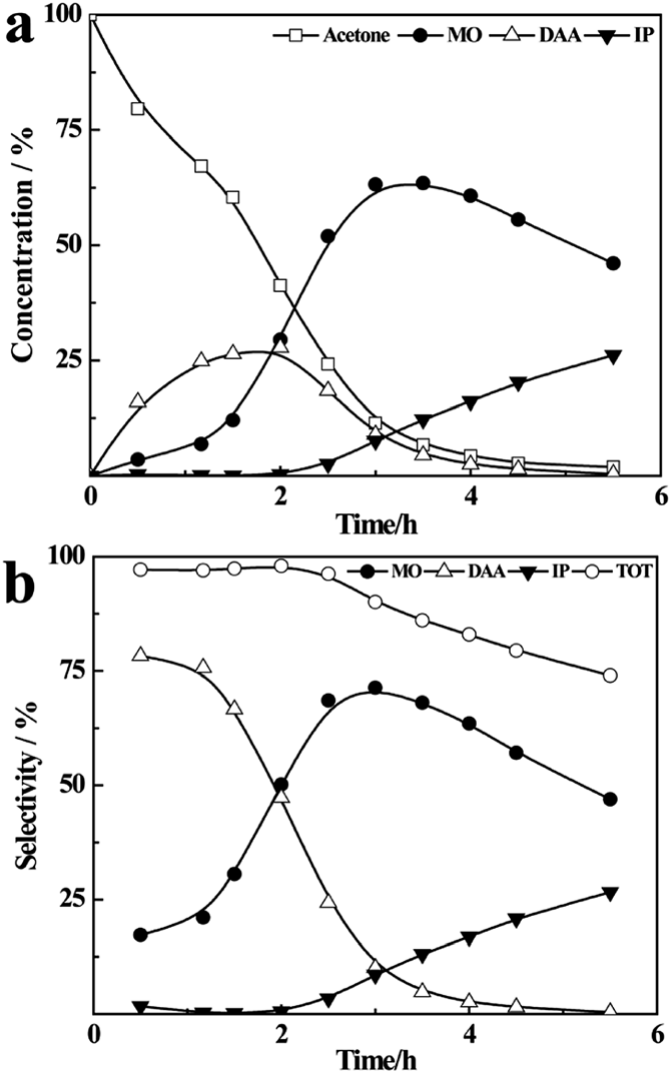
The concentration (a), selectivity (b) at higher temperature (∼90–115 °C).

As shown in Fig. 3, in the initial 1.5 h, more than 55% of acetone is converted to DAA and MO, and the selectivity of DAA is higher. After that, the content of DAA decreases quickly along with a drastic increase of MO from 12% at 1.5 h to 63% at 3 h, due to the dehydration of vaporized DAA at higher temperatures on the surface of the catalyst. From third hour onwards, the concentration of MO decreases with time due to its further reaction with acetone on the catalyst, which results in an increasing content of IP. Meanwhile, the amount of oligomers also increases with time, which leads to a decreased total selectivity of DAA, MO and IP from 97% to 73%, see Fig. 3(b). Compared with the reaction results at 56 °C, it is obvious that higher temperature is favourable to the yield of MO and IP, and the formation of MO is a kinetically controlled process.^41^

### Influence of reflux rate on product selectivity

**Fig. 4:**
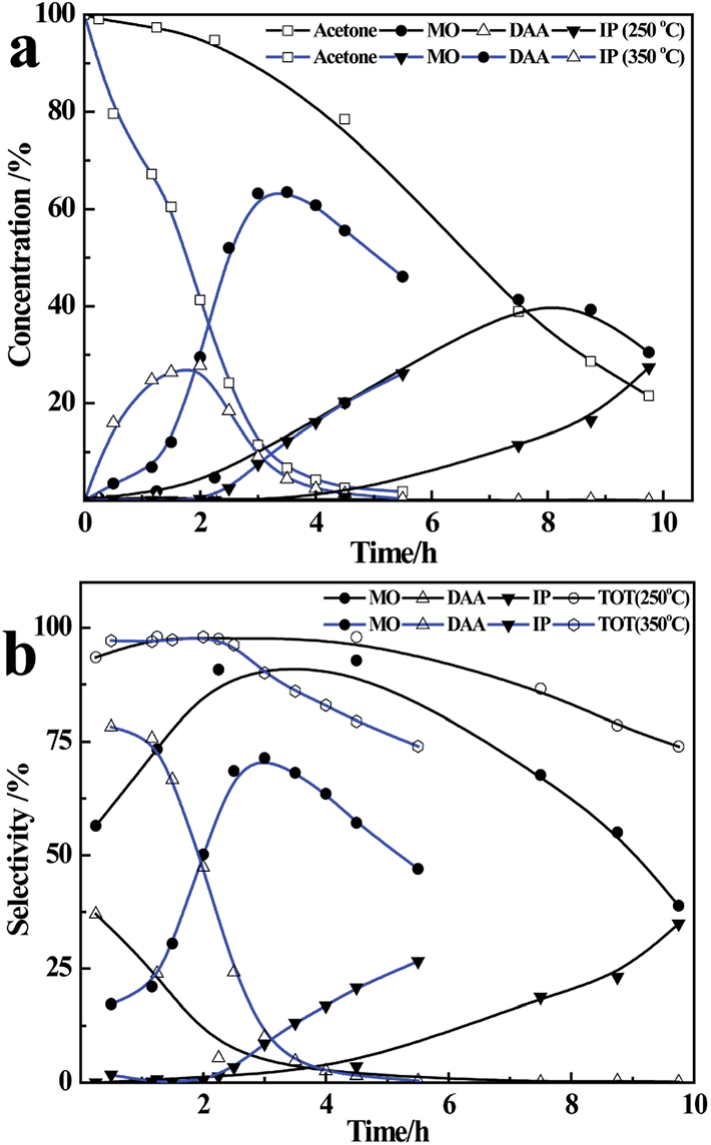
Variation of liquid concentration (a) and selectivity (b) with time at two reflux rates.

To conduct the reaction at a lower reflux rate, the flask was heated at 250 °C instead of 350 °C, while other conditions remained the same as above. The reaction results are presented in Fig. 4. Note that the reflux rate has a substantial influence on the reaction rate and product selectivity. As the heating temperature decreases from 350 °C to 250 °C, the conversion of acetone at 4 h decreases significantly from 95% to 23%, and the main products become MO and IP with negligible amounts of DAA. This is because lower reflux rate means a lower liquid–solid mass transfer rate,^42,43^ and hence a lower conversion of acetone. Besides, with a longer residence time on the catalyst, DAA tends to be dehydrated to MO. As a result, the yield of DAA becomes negligible at lower reflux rate or lower heating temperature accompanying a very high selectivity of MO, *i.e.* 90% in initial 4 h, as shown in Fig. 4(b). After 4 h, the selectivity of MO declines gradually due to its further condensation with acetone on the catalyst, resulting in an increased yield and selectivity of IP. However, the total selectivity of DAA, MO and IP decreases gradually from 95% at 4 h to 75% at 10 h due to increased amount of oligomers. In short, the reflux rate has a remarkable influence on the condensation of acetone; higher reflux rate is favourable for the formation of DAA and MO, and a lower reflux rate is favourable for the formation of MO, IP and more oligomers.

### Industrial process design

As shown above, the reaction rate and product selectivity can be well controlled by temperature and instant separation of the acetone derivatives from the catalyst. The reaction temperature can be adjusted by the operating pressure, which is mainly determined by the vapour pressure of acetone. According to the Antoine equation, the vapour pressure of acetone at 90 °C and 115 °C is 280 and 530 kPa, respectively, which is in the low pressure range and thus is applicable for industrial catalytic distillation.

**Fig. 5:**
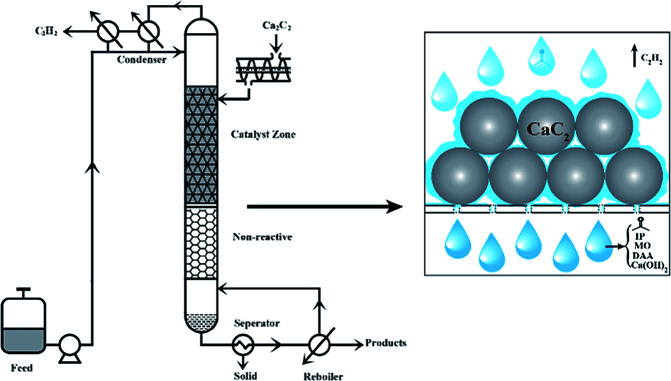
Industrial process design.

As we know, lower temperature is advantageous for the selective production of DAA and MO, whereas higher temperature favours the formation of MO and IP, and higher reflux is beneficial for the instant separation of DAA, MO and IP from the catalyst so as to avoid the formation of oligomers. The optimal conditions depend on the target products, which can be tuned effectively in a distillation column. Based on the above recognition, a schematic process is proposed, as shown in Fig. 5. The distillation column is composed of two parts, the reaction zone of the upper part with CaC_2_ granule as the catalyst and packing, and the distillation zone with sieve plate or ceramic packing. Assuming that the liquid mixture in the reboiler is an ideal solution with 20% acetone, 44% MO, 32% DAA and 4% IP, the temperature in the reaction zone is 56 °C, and all products in the reboiler will not go to the reaction zone, at least 2 theoretical plates are required for the distillation zone, and the boiling temperature of the reboiler is about 95 °C. If a higher selectivity of MO and IP is required, we can raise the reaction temperature or reduce the reflux rate.

The operation process can be illustrated as follows. The acetone feedstock is pumped to the condenser for preheating and then enters to the top of the reaction column at a specified flow rate. In the upper part of the column, aldol condensation and dehydration reactions take place on the surface of CaC_2_ catalyst, forming DAA, MO, IP, acetylene gas, and Ca(OH)_2_. The acetylene gas goes up to the condenser and exits the reaction system as tail gas. The as-formed Ca(OH)_2_ nanoparticles are constantly washed away by the down-flowing liquid (DAA, MO, IP, acetone, and other heavy components, if any) to the reboiler due to its much smaller size than the passage of packing and sieve holes of the support. The slurry liquid mixture from the reboiler can be easily separated to Ca(OH)_2_ and the product mixture by filtration or centrifugal settling due to the much higher density of Ca(OH)_2_ than that of the liquid mixture. The fresh CaC_2_ can be refilled into the reaction zone by a mechanical screw batch charger. As such, a novel and efficient process for the reaction of CaC_2_ and acetone is achieved for the coproduction of DAA, MO, IP, and acetylene with high selectivity and yield. This process seems superior to the conventional ones considering the much higher catalytic activity of CaC_2_ than all the catalysts reported,^26^ high conversion rates and selectivity under mild conditions, and accordingly lower capital and running cost. Furthermore, the catalyst cost is negligible, since we only use the super-basic catalysis of CaC_2_ without sacrificing its original value for the production of acetylene.

## Conclusion

For the efficient synthesis of DAA, MO and IP, we proposed a novel reaction process of refluxing acetone on CaC_2_ surface. With this process, the high catalytic activity of CaC_2_ can be used effectively, and the target products can be instantly removed from the catalyst so as to avoid their further conversion to oligomers, leading to an efficient and highly selective production of DAA, MO and IP. Further, the product selectivity and distribution can be finely tuned by the reaction temperature and reflux rate of the distillation column. Lower temperature and higher reflux rate favour the production of DAA and MO, otherwise MO and IP are favoured. Therefore, the present process realizes an excellent control of product selectivity for the condensation of acetone, and may be viable for the high value uses of acetone and coproduction of C_2_H_2_.

## Conflicts of interest

There are no conflicts to declare.

## Supplementary Material

RA-008-C8RA05965A-s001
