# Supplementary material for: Aldol condensation of refluxing acetone on CaC2 achieves efficient coproduction of diacetone alcohol, mesityl oxide and isophorone
Source: RSC Adv. 2018 Aug 30;8(53):30610–5. doi: 10.1039/c8ra05965a (PMC9085500; doi:10.1039/c8ra05965a)
Supplement: RA-008-C8RA05965A-s001 [file RA-008-C8RA05965A-s001.pdf]

Electronic Supplementary Material (ESI) for RSC Advances.
This journal is © The Royal Society of Chemistry 2018

Aldol condensation of refluxing acetone on CaC_2 achieves efficient coproduction of diacetone-alcohol, mesityl oxide and isophorone

Xuebing Xu[a,b], Hong Meng[b], Yingzhou Lu[b], Chunxi Li[a,b,c,]*

[a] State Key Laboratory of Chemical Resource Engineering; [b]College of Chemical Engineering, [c] Beijing Key Laboratory of Energy Environmental Catalysis, Beijing University of Chemical Technology, Beijing 100029, PR China; Tel/Fax: 86-10-64410308; E-mail: Licx@mail.buct.edu.cn

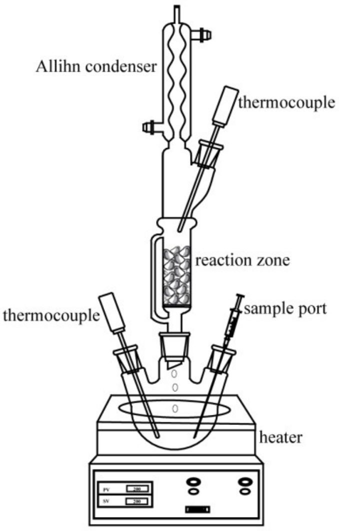

Fig.S1 Refluxing reaction experimental setup

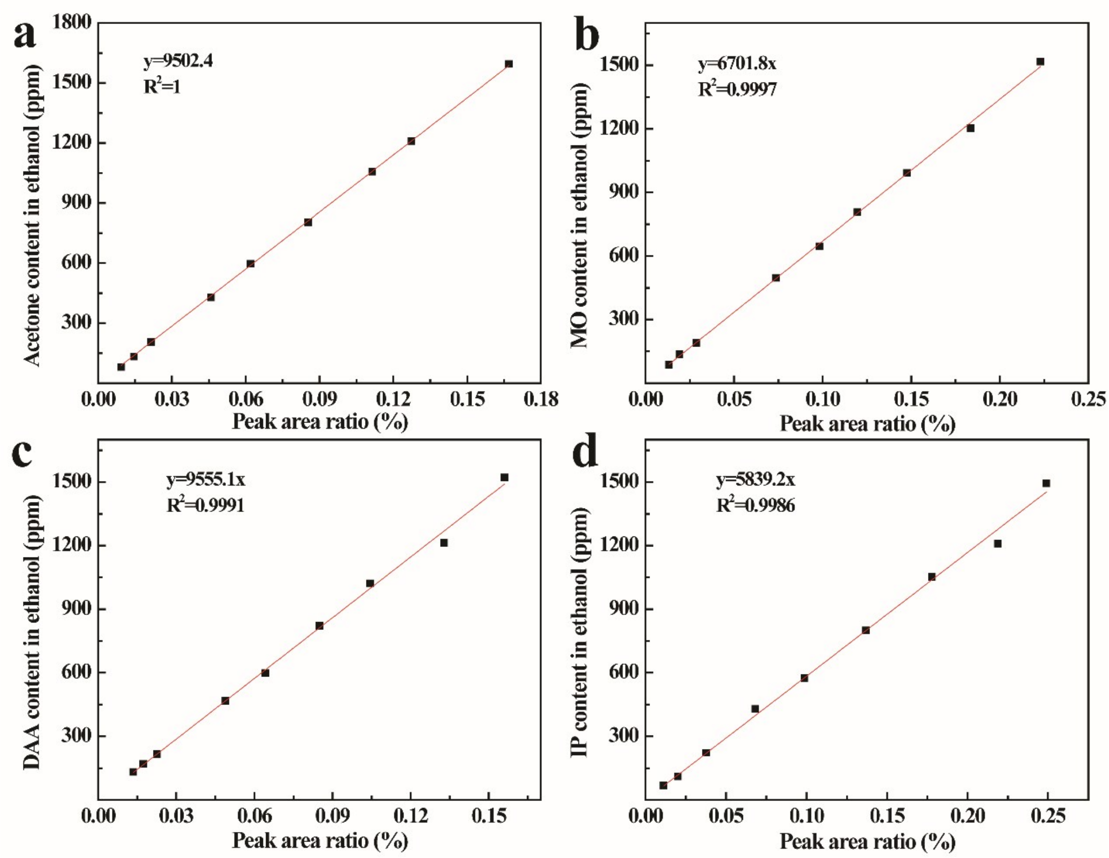

Fig.S2 Standard curve of acetone-ethanol (a), MO-ethanol (b), DAA-ethanol (c) and IP-ethanol (d)

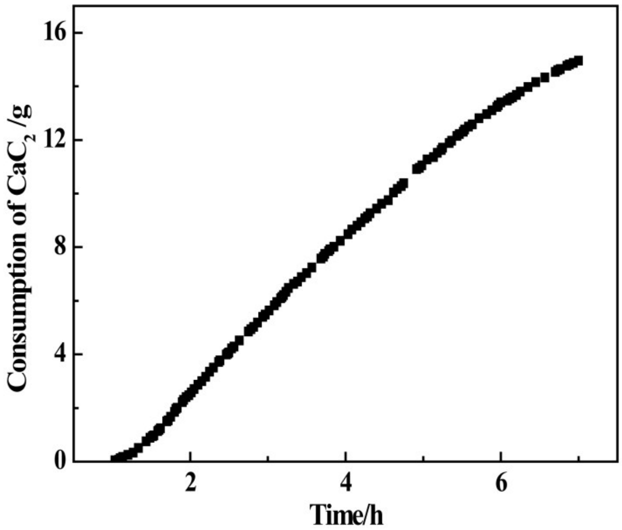

Fig.S3 Consumption of CaC_2 at the boiling point of acetone

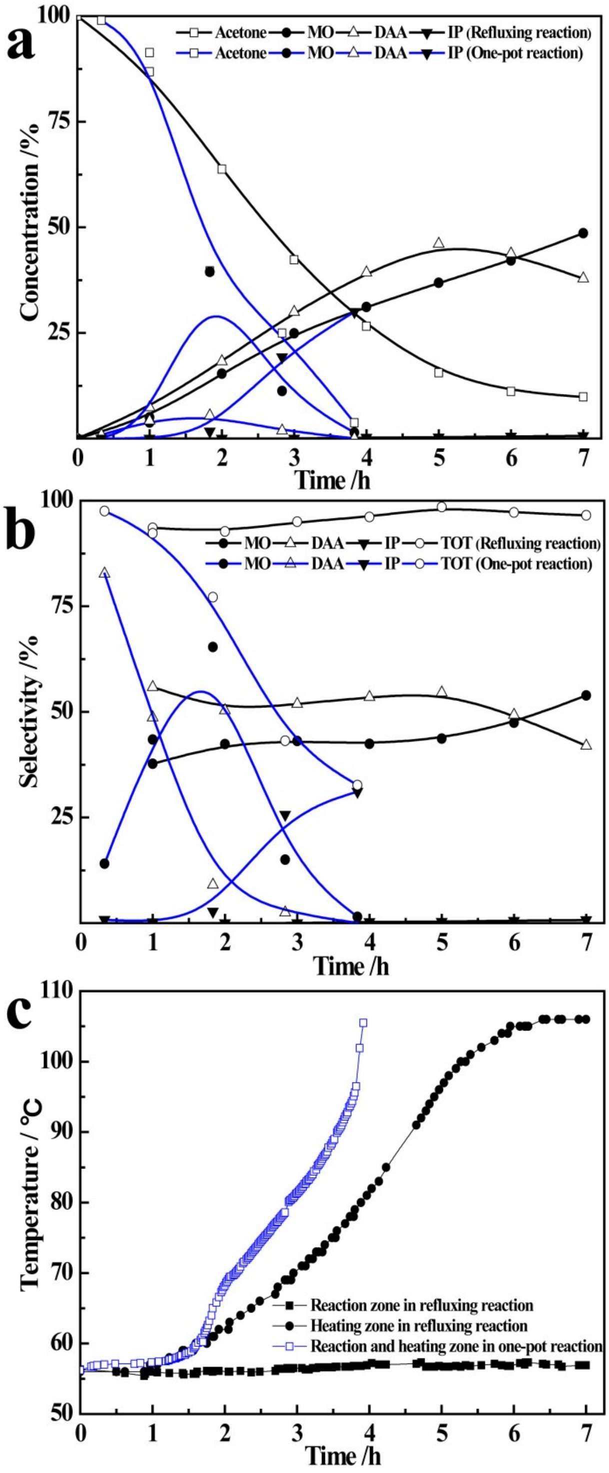

Fig. S4 Variation of liquid concentration (a), selectivity (b) and temperature (c) with time at two reaction processes
